# A Qualitative Analysis of How Online Access to Mental Health Notes Is Changing Clinician Perceptions of Power and the Therapeutic Relationship

**DOI:** 10.2196/jmir.6915

**Published:** 2017-06-14

**Authors:** Lauren M Denneson, Risa Cromer, Holly B Williams, Maura Pisciotta, Steven K Dobscha

**Affiliations:** ^1^ VA Portland Health Care System Center to Improve Veteran Involvement in Care (CIVIC) Portland, OR United States; ^2^ Oregon Health & Science University Department of Psychiatry Portland, OR United States

**Keywords:** eHealth, physician-patient relations, mental health, patient-centered care

## Abstract

**Background:**

As part of the national *OpenNotes* initiative, the Veterans Health Administration (VHA) provides veterans online access to their clinical progress notes, raising concern in mental health settings.

**Objective:**

The aim of this study was to examine the perspectives and experiences of mental health clinicians with OpenNotes to better understand how OpenNotes may be affecting mental health care.

**Methods:**

We conducted individual semi-structured interviews with 28 VHA mental health clinicians and nurses. Transcripts were analyzed using a thematic analysis approach, which allows for both inductive and deductive themes to be explored using an iterative, constant comparative coding process.

**Results:**

OpenNotes is changing VHA mental health care in ways that mental health clinicians perceive as both challenging and beneficial. At the heart of these changes is a shifting power distribution within the patient-clinician relationship. Some clinicians view OpenNotes as an opportunity to better partner with patients, whereas others feel that it has the potential to undo the therapeutic relationship. Many clinicians are uncomfortable with OpenNotes, but acknowledge that this discomfort could both improve and diminish care and documentation practices. Specifically, we found that (1) OpenNotes is empowering patients, (2) OpenNotes is affecting how clinicians build and maintain the therapeutic relationship, and (3) mental health clinicians are adjusting their practices to protect patients and themselves from adverse consequences of OpenNotes.

**Conclusions:**

Our findings suggest that future research should monitor whether OpenNotes notes facilitates stronger patient-clinician relationships, enhancing patient-centered mental health care, or diminishes the quality of mental health care through disruptions in the therapeutic relationship and reduced documentation.

## Introduction

Health care systems across the United States are beginning to allow patients to view their electronic health records, including clinical progress notes, online. These *OpenNotes*, and the OpenNotes initiative more generally [1], respond to recent legislation calling to increase patients’ access to their health information [2-4] and are intended to improve health care transparency, facilitate patient sharing of health information with other clinicians, and encourage patient engagement in health care. The few available studies show that patients who read their medical record progress notes are more satisfied with their care, feel more informed about their health, and are more engaged and adherent to care [5-8]. Before a national rollout in 2013, the Veterans Health Administration (VHA) piloted OpenNotes at several sites across the United States to examine patient, clinician, and system-level outcomes [6]. Findings were favorable, with the majority of patients saying that OpenNotes helps them understand their health history and conditions, manage their health, prepare for clinic visits, and take their medications as prescribed [9,10].

Unlike other health care systems implementing OpenNotes, the VHA does not provide note writers the option of preventing selected notes from becoming available to patients online. As such, veterans can read or download any of their VHA progress notes (written after 2013) online, including progress notes detailing their mental health care. This removes logistical barriers for patients who wish to read their mental health notes. Although patients have had the legal right to request paper copies of their medical records for some time [11], the process can be cumbersome and time-consuming, resulting in few patients typically exercising this option [12]. Furthermore, since mental health notes often contain sensitive information about patients’ mental illness, they have historically been treated differently from other progress notes, sometimes requiring clinician approval for patients to see or receive paper copies of their notes [13]. Despite promising findings from studies of primary care patients [9,10], some are concerned that this increased ease of access to mental health notes may cause unnecessary worry, confusion, or distress among patients who read their mental health progress notes without guidance or permission from their clinicians [14,15].

In a brief survey of VHA mental health clinicians about OpenNotes, approximately half of clinicians did not feel that mental health OpenNotes was a good idea [14]. Although they thought some positive outcomes might come out of OpenNotes, most expressed concern over potential negative consequences from OpenNotes and reported making changes to their note writing practices, including writing fewer details, changing the tone of the note, and writing less information about diagnoses [14]. However, the impetus for such concern and documentation changes is poorly understood. In this study, we use qualitative methods to further examine mental health clinicians’ perspectives on and experiences with OpenNotes, to better understand how mental health clinicians approach care and documentation in the context of OpenNotes.

## Methods

### Setting and Sample

We conducted this qualitative study at a VHA Medical Center that provides comprehensive care at 11 urban and rural sites. Over 250 mental health clinicians (psychiatrists, psychologists, social workers, nurse practitioners) and nurses (registered nurses and licensed practical nurses) provide mental health care to approximately 18,000 unique patients each year across a variety of services spanning inpatient care to homelessness programs. “MyHealtheVet” is the VHA’s online patient portal through which veterans receiving VHA care can access their health care records and progress notes, refill prescriptions, and securely email their clinicians.

All clinicians and nurses providing mental health care at any of the medical center’s clinic sites were eligible for study participation. We sent recruitment emails to all eligible staff to describe the purpose of the study and invite interested staff to contact the study team. A total of 28 clinicians and nurses were interviewed between May and October 2014; enrollment was halted when the study team agreed we had reached saturation of themes. Over half (16/28; 57%) of the participants were female, and participants represented a range of disciplines: social workers (10/28; 36%), psychiatrists (7/28; 25%), psychologists (5/28, 18%), mental health nurse practitioners (3/28; 11%), and nurses (3/28; 11%). Participant’s length of time working within VHA ranged from 1 to 30 years (mean 11.1 years).

### Data Collection

We developed a semistructured interview guide informed by the main research questions and aims of the project, current literature on patient experiences with full health record access [7,16,17], and input from mental health clinicians. The interview guide focused on elucidating clinicians’ thoughts across four main domains: (1) general knowledge and attitudes about OpenNotes, including familiarity with OpenNotes, concerns, and benefits; (2) experiences discussing OpenNotes with patients, including responding to patient concerns or initiating conversations; (3) experiences and changes in documentation; and (4) recommendations to other clinicians and education needs regarding clinical practice in the context of OpenNotes. For this analysis, we focused on the first three interview domains. All interviews were conducted in person by nonclinicians with backgrounds in public health and anthropology, and each interview lasted approximately 60 min. Interviews were transcribed and validated for accuracy by an independent reviewer.

### Data Analysis

We used ATLAS.ti software (ATLAS.ti Scientific Software Development GmBH) to organize transcripts and facilitate analysis. We used a thematic analysis approach [18,19], which allows researchers to bring preexisting research questions to their analysis of the data while also investigating entirely unanticipated themes. Thus, deductive and inductive codes, respectively, were identified and used in our analysis. We used the main topics from our interview guide to create initial, deductive codes. Then, through an iterative, open coding process, three analysts (RC, HBW, and MP) reviewed transcripts to identify themes emerging from the text to create inductive codes. Together, these codes comprised our codebook. Once all three analysts agreed that the codebook contained the themes emerging from the transcripts, the codebook was considered final. Then, after a calibration period, two analysts applied the codebook independently to all transcripts, with a third analyst arbitrating, as needed. Specific text passages relating to the codes were compiled into code reports for analysis. All authors then reviewed and discussed code reports for thematic interpretations to refine main themes, resulting in our primary findings. In the results presented here, we use participant quotes to illustrate our findings, which are labeled using a numeric code (eg, 1001).

## Results

### Overview

(OpenNotes) really is antithetical to the way that many of us have been, literally, trained and learned to think about our field.

1008

Overall, analyses revealed that OpenNotes is changing VHA mental health care in ways that mental health clinicians perceive as both challenging and beneficial. At the heart of these changes is a perception of shifting power distribution within the patient-clinician relationship; OpenNotes provides patients with easier access to information about their health, their health care, and clinicians, resulting in more equitable distribution of power between clinician and patient. This is affecting how clinicians are navigating the therapeutic relationship and making changes to how they practice. Rapport building, which typically relies on carefully delivered conversations to help the patient feel comfortable enough to engage in the therapeutic relationship, is a key concern for clinicians, as progress notes leave room for miscommunication and misinterpretation. Many clinicians are uncomfortable with OpenNotes and want guidance on how to adjust their practices to protect patients and themselves from adverse consequences of OpenNotes. In the paragraphs that follow, we elaborate on these key themes: (1) OpenNotes is *shifting the patient-clinician power distribution*; (2) OpenNotes is affecting how mental health clinicians build and maintain the *therapeutic relationship* and therapeutic process; and ultimately, (3) mental health clinicians are *adjusting their practices in the context of OpenNotes* to protect patients and themselves from potential adverse consequences. Selected quotes illustrate these themes (see Multimedia Appendix 1).

### Shifting Patient-Clinician Power Distribution

Clinicians discussed having less control over when and where patients access information contained in progress notes; online access to progress notes was thought to provide patients with more information about their health as well as an increased level of transparency about their health care. Clinicians compared their previously high level of control over the release of information in patients’ medical records with their current lack of control (when patients read their notes online, clinicians are not informed). Some clinicians embraced this, whereas others wished to regain some control.

Yes it definitely has changed. There was a definite time we used to get a...message saying “can this person read their own record?”

1009

Many clinicians perceived the increase in patient access to, and resultant control over, their health information as a change in the distribution of power between patients and clinicians, with power becoming more equally distributed. Clinicians were not necessarily concerned about the loss of power per se, but about how the power shift affected their approach to providing care. Some clinicians viewed this shifting power differential as a move in the right direction, toward “patient-centered” care, creating better opportunities for collaboration with patients and facilitating patient engagement in care.

Basically, it lessens that knowledge gap between the treatment team and the patient in terms of what it is we’re working towards and how does the treatment plan go about trying to achieve these goals.

1029

On the other hand, a few clinicians described feeling that patients could use OpenNotes to dictate to clinicians on how to write their notes and—by extension—direct their care. These clinicians were often referring to a small portion of patients that they described as particularly challenging, such as those diagnosed with borderline personality disorder or schizophrenia.

What I’m noticing is that, and I’ve directly had patients say this to me, “...don’t write that in my notes.” ...It’s just like they’re trying to dictate their care and we’re trying to provide care...I feel like I’m on the defense.

1016

### Therapeutic Relationship

Clinicians discussed the idea that developing good rapport and a therapeutic relationship is critical to patient engagement and recovery in mental health care. However, this process can be difficult and requires careful work on the part of clinicians to earn patients’ trust.

A lot of times with mental health, there is sort of a dance that’s done where a patient comes in, drops out and comes in and drops out again, and then finally comes in and feels safe enough and trusts enough to get the help. Anywhere along that line the trust gets hurt, that could be it and they are never seen again. We know that there is a lot of untreated mental illness for a lot of reasons, but that’s certainly one. It’s very hard to trust people with your most near and dear emotional psychological stuff. Trust is just the main thing we’ve got to help people in the mental health field, and so that’s my real concern is that we run the risk of damaging trust with our patients.

1008

Many clinicians expressed concern that providing patients easier access to their notes could damage the therapeutic relationship by exposing a disconnect between the patients’ in-person experience with their clinicians and the documentation they read in their notes. Specifically, notes reveal aspects of the therapeutic process—such as clinical formulations and subjective impressions—which clinicians frequently do not communicate to their patients. As such, reading notes could create opportunities for patients to negatively misinterpret clinicians’ notation, or increase the likelihood that patients feel judged, stigmatized, or otherwise looked poorly upon by their clinicians. Some clinicians felt that notes had the potential to undo the work they did in session to develop good rapport with their patients.

People can feel belittled about something. I had somebody come in not too long ago, within the last few months, saying in a really angry way that “I don’t see what my haircut has to do with anything.” That’s part of the mental status exam. Obviously appearance, grooming and hygiene are something we attend to see about a person’s depression and their hygiene and how are they taking care of things. He felt very criticized by that. I don’t think he feels criticized when he’s here with me. But reading that caused a separation that I think might not have been disturbing to him if he had not seen that in print.

1008

On the other hand, some clinicians saw potential for OpenNotes to benefit the therapeutic relationship by enhancing feelings of trust and transparency, providing opportunity for enhanced communication even when there are disagreements, and showing patients that they are listening and have patients’ best interests in mind.

I’ve heard that a couple of times, “from your charting I could see how much work you’ve put into it, and I could see that you care about me, and the plans that we come up with—you are hearing the things I want out of our goals and plans.”

1027

### Adjusting Practice in the Context of OpenNotes

Mental health clinicians described being careful about what they write in progress notes as a matter of course; they are acutely aware of the clinical, legal, and other audiences of notes within a large integrated health care system that uses a common electronic medical record. However, they expressed increased discomfort with the added complexity of writing notes that their patients can access online, keeping in mind both realized and potential benefits and adverse effects of patients reading their notes.

There’s another one where someone said, “I smoked meth for 40 years and my wife doesn’t know.” And I was like, gosh, do I put this in the note? Because I don’t know if he is going to give his wife access to his notes and then see something that was delivered in confidence...

1006

Often we’re taught to document things in a particular way in order to cover ourselves for legal concerns and adding the layer of actually having the client also reading these notes just adds an additional layer of complexity to what you have to think about and how you have to phrase things in your documentation.

1005

In particular, clinicians felt a strong desire to protect their patients from potential harms, while also feeling vulnerable and exposed themselves.

Then again, for me, the onus is on us. We’re the ones who are responsible for creating safety. I think that’s a big part of this. If OpenNotes were to trigger somebody or create a safety issue, it’s still on us to do our best to resolve it in a safe way. It shouldn’t be on the person who is sick or war-torn to navigate it.

1011

So I just feel like it hinders my ability to work without the feeling—sometimes I feel a little threatened, I feel there is going to be really negative consequences if I write what I’m assessing to be clinically accurate.

1016

Some clinicians felt this discomfort functioned to help keep clinicians accountable, and would ultimately result in improved care and documentation. Furthermore, some clinicians liked that patients can now review notes and point out inaccuracies, which was viewed as another way to increase clinician accountability and improve the medical record documentation.

I think it has this sense of increasing empathy on my part. I really try to see where people are at. And I think when they’re coming in here saying, “this hurt, this is what’s written in my record,” it forces us to kind of be in their shoes a little bit when you know they’re clicking on that button and seeing what you wrote.

1023

When you know that other people are looking at the work that you do, particularly the people who it directly pertains to, then you want to make sure it’s the best, it’s the most accurate.

1023

Many clinicians were hoping for system-level guidance on how to best document care to reduce the potential for negative outcomes for them or their patients.

I would appreciate some clarity on who the audience is, on who I’m writing for. And I think in general, training in this more recovery-oriented and strengths-oriented treatment in general. Moving away from thinking about things, like in the medical model, in terms of problems and thinking about things more as this being a collaborative relationship with their clients.

1005

Some sort of agency/VA-type guidance on what’s expected to be in a note. What should and shouldn’t be left out in order to minimize the risk of the open note problem, or the potential open note problem...

1008

Without such guidance, some clinicians described making their own adjustments to documentation—writing fewer details, using vague terms, striving for increased objectivity, or adjusting how they document patient quotes (some increasing use of quotes and some decreasing)—as well as holding proactive conversations with patients about their notes.

How have I adapted? My notes are a lot less detailed now, here. I always have to kind of couch what I’m saying. There’s much less detail, much less frankness in my notes now

1006

I do an informed consent about (OpenNotes). I think it’s dangerous, I tell them, “Look, there’s this thing called the blue button. You’ll hear about it. You may want to push it. If you do you’re going to see your clinical notes. That’s fine by me, but understand there’s stuff here that I’m going to write what I hear and see and it may be upsetting to you, and you may or may not want to do it, but there’s risks associated with it.”

1010

## Discussion

Overall, we found that OpenNotes within VHA is affecting how mental health clinicians think about their relationships with patients and the progress notes they write. Primarily, they perceived reduced control over the flow of information pertinent to the therapeutic process; the notes they write are now accessible to patients at any time without clinician approval or other barriers. This change necessarily shifts the patient-clinician power dynamic toward a more equitable distribution.

Interestingly, we found that some clinicians described this power shift as a move toward more “patient-centered” care. Patient-centered care is often characterized by shared patient-clinician power and responsibility, a biopsychosocial orientation, patient and clinician humanity, and a therapeutic alliance [20]. True patient-centered care has been thought to be difficult to implement in mental health settings, where clinicians historically have had role expectations in which the patient is viewed as someone to “protect” and for whom the clinician is “responsible” [21-23]. We saw similar themes in this study. Many clinicians felt that OpenNotes provided benefits such as enhanced opportunity for collaboration, mutual trust, and addressing patients’ concerns. However, many were also concerned that OpenNotes could cause unintentional harm for their patients—for whom the clinicians would feel responsible. Indeed, mental health clinicians’ changes in note writing reflected a desire to write notes that would mitigate potential harm. This concern could be viewed as stemming from clinicians feeling obligated to protect their patients in the more traditional, paternalistic style of care. In previous work, nearly two-third of VHA mental health clinicians surveyed stated that they had made changes in how they document as a result of OpenNotes, with the majority reporting that they write fewer details in the notes [14]. Together, this suggests that while OpenNotes may help to facilitate care that is more aligned with patient-centered care ideals, mental health clinicians are also often limiting what they write in response to a desire to protect themselves and their patients, which could have unintended negative consequences such as forcing clinicians to rely on their memory more often or reducing clinician-clinician collaboration.

Although most clinicians felt some discomfort with OpenNotes, it is also important to note that some clinicians thought this discomfort might improve care by motivating clinicians to be at their best and providing an impetus to generate “difficult” but important conversations between patients and clinicians. Clinicians surmised that documentation could become more accurate as clinicians pay closer attention to what they write and patients have the opportunity to review and request changes. Generally, clinicians wanted guidance to help them navigate how to document in the context of OpenNotes, suggesting that mental health clinicians are motivated to improve and adapt their documentation.

Our findings should be considered in light of several limitations. We interviewed clinicians and nurses who expressed interest in participating; they may have stronger concerns or views than other clinicians. However, we heard a wide range of opinions and thoughts in response to our questions and we heard a range of experiences in the extent to which clinicians talked about OpenNotes with their patients and their knowledge of patients using OpenNotes. We also note that we do not know the actual potential for the possible outcomes of OpenNotes that clinicians discuss here (eg, diminished care due to reduced documentation). This study was conducted at one VHA medical center in the Pacific Northwest; views on OpenNotes may differ across other regions of the United States or at other VHA facilities. Finally, the VHA is unique in ways that may affect how mental health clinicians think about documentation, limiting our findings’ generalizability to other health care settings. For some examples, VHA is an integrated care system in which nonmental health clinicians have access to their patients’ mental health notes; some patients receive health benefits as a result of injury during military service, which can be inadvertently affected by clinician documentation; and some veterans eligible for redeployment may worry that contents of their medical record could impact redeployment eligibility.

Findings from this study suggest that online patient access to their mental health progress notes is changing how VHA mental health clinicians think about and document care—in both positive and negative ways. Clinicians perceive a shift in the balance of power between clinicians and patients, primarily resulting from reduced clinician control over the flow of information pertinent to the therapeutic relationship and process. Clinicians often view this as a shift toward more patient-centered care, but many find the change uncomfortable. This discomfort may result in improved documentation and conversations with patients, or could lead to some unintended negative consequences such as reducing what they document. Future research should continue to monitor impacts of OpenNotes in mental health settings and identify methods to reduce potential harms and enhance benefits of OpenNotes.

## Appendix

Multimedia Appendix 1 Main themes and supporting clinician interview quotes.

## Abbreviations

VA: Veterans Affairs
VHA: Veterans Health Administration
