# Supplementary material for: A Qualitative Analysis of How Online Access to Mental Health Notes Is Changing Clinician Perceptions of Power and the Therapeutic Relationship
Source: J Med Internet Res. 2017 Jun 14;19(6):e208. doi: 10.2196/jmir.6915 (PMC5489707; doi:10.2196/jmir.6915)
Supplement: Multimedia Appendix 1 [file jmir_v19i6e208_app1.pdf]

Multimedia Appendix 1. Main themes and supporting clinician interview quotes.

**Shifting patient-clinician power distribution**	
Patients can control if and when they read their notes, without asking permission.	Yes it definitely has changed. There was a definite time we used to get a… message saying 'can this person read their own record?' In an ideal world the person would come to us and we could say, 'Please, you may find words or language in there that is confusing to you; don't hesitate to give us a call, we'd love to talk this over with you if you have anything - some feelings may come up for you reading what clinicians have written about you.' (1009)
	And then I keep going back to, you know, the whole thing of, who makes the decision of which veteran is mentally capable of dealing with the information they see in the notes. And then again, to say that no one should be able to see that information, I don't like that either. So either they see everything or nothing, that's how I feel. I don't think it should be done in piecemeal. (1030)
	Joking aside, a lot of people really perceive a huge power differential, lack of respect, lack of caring, lack of empathy, lack of listening. I'm glad that there's nothing that can be excluded [from OpenNotes]. I hope there is no OpenNotes exclusion flag in the future popping up. 'Borderline. Totally made issue with notes. Let's flag them.' If that happens, I think that it will be a sad day but I'm sure it won't. (1011)
	I think the clinician having the option to be the one to give the note to the veteran, in a timely way, I think that would be a reasonable thing, a buffer to add in. Maybe that would make everybody happy. (1018)
Increased patient control over access to information changes power distribution between patients and clinicians.	Basically, it lessens that knowledge gap between the treatment team and the patient in terms of what it is we're working towards and how does the treatment plan go about trying to achieve these goals. (1004)
	You get opportunities for a better collaboration and partnership between patients and providers. You have healthcare decision making, as a result of this access, moving in the direction of a patient centeredness. As opposed to a clinician having all of the information. (1020)
	…the relationship between the veteran and providers should always have a goal of being a partnership. I really try to strike down the power imbalance between the veterans and how they see the VA, and how they see the providers – understand that our goal is not to judge them or make life difficult, but to do what we can to the best of our abilities to provide for them. That having given you access to your notes should be looked at as our efforts of trying to be more transparent. That there is nothing to be afraid of, that this not a big system out to get you. (1014)
	…to see that available to veterans I think is wonderful and I think it really empowers the veteran to have more actual – they can advocate for their healthcare and they can be very actively participating instead of the more traditional, you know, doctor-patient sort of dynamic. I think the dynamic is changing more in society in general, so it's really interesting to see the VA come in with that. (1029)
Certain patients could exert their power to dictate note content and care.	What I'm noticing is that, and I've directly had patients say this to me, '…don't write that in my notes.' …It's just like they're trying to dictate their care and we're trying to provide care…I feel like I'm on the defense. (1016)
	It would get into this dynamic where they would want to dictate what was going in

	the chart notes. They would want to spend the session telling the providers, 'Okay. I want you to put this in the note and not that in the note. I want you to use words and not those words.' …I feel like it can be unintentionally turned into a tool to kind of harass providers in certain cases by veterans who are particularly perseverative about the notes. (1026)
	I think typically there's this power struggle and 'we are the expert and you are coming to us for help.' I think a lot of people that I work with work hard to decrease that power gap and include veterans…Now the veteran has some power that they didn't before. I think that can be scary and a vulnerable place, and without maybe having the skills to know how to combat them that can also be stressful for the provider. Depending on the population you work with I think – I think about a lot of clients who might have obsessive behaviors or personality traits that could be problematic around arguing with the clinician. Different dynamics like that might come up that add a little bit more stress or concern for the clinician. (1013)
	Let's see like the borderline personality disorder, oftentimes there's a lot of power struggle at baseline. And so when a person comes in and they're acute and they might run a course of several days that are, it's pretty volatile; lots of behavior problems and limits and, you know, documenting some of those behaviors, which you want to do, in your notes, I could maybe see the potential of being a power struggle or the splitting, you know, that is kind of characteristic to that particular group of patients. (1029)
**Therapeutic Relationship**	
Therapeutic relationships are critical to mental health care, but can be a challenge to develop.	A lot of times with mental health, there is sort of a dance that's done where a patient comes in, drops out and comes in and drops out again, and then finally comes in and feels safe enough and trusts enough to get the help. Anywhere along that line the trust gets hurt, that could be it and they are never seen again. We know that there is a lot of untreated mental illness for a lot of reasons, but that's certainly one. It's very hard to trust people with your most near and dear emotional psychological stuff. Trust is just the main thing we've got to help people in the mental health field, and so that's my real concern is that we run the risk of damaging trust with our patients. (1008)
	I think clinicians are really wary of it because it's kind of, as a clinician, a very vulnerable place to be, so we're not used to – at least after training – we're not used to people having that window into what we do. (1013)
Certain information, misunderstandings, or the tone of progress notes could damage the therapeutic process and relationships with patients.	Or, people can feel belittled about something. I had somebody come in not too long ago, within the last few months, saying in a really angry way that 'I don't see what my haircut has to do with anything.' That's part of the mental status exam. Obviously appearance, grooming and hygiene are something we attend to see about a person's depression and their hygiene and how are they taking care of things. He felt very criticized by that. I don't think he feels criticized when he's here with me. But reading that caused a separation that I think might not have been disturbing to him if he had not seen that in print. (1008)
	What's lost – I think there's a loss of mystique, the mystery, the idea that what a patient should really know about their treatment is that they come in and what they see happening in the room, if they have good feelings, if they get good suggestions and things like that. I think when somebody reads the notes it's essentially pulling the curtain and seeing what's really there, and I think it damages that process…In

	saying 'hey there are attachment issues, difficult relationship with mother and abuse, has harmed his ability to connect to his wife in this way.' It's that assessment that usually in psychotherapy you want to help the patient come to that conclusion on his or her own. You don't just say 'here's the script and here's the ending' I think it interferes with that process. (1006)
	Some of our folks have pretty powerful delusions. When you're aligning with somebody, sometimes you don't challenge those delusions because you're trying to continue to build that rapport. So there's that piece. (1020)
	I work with a veteran that does not believe he has a mental illness. In his mind, we are treating him for stress and sleep issues. He's been very reluctant to take medications and is taking medications, including an antipsychotic, that also helps with sleep. So we're reselling it to him as a sleep aid and as something that will help with his anxiety. But it is an antipsychotic. (1005)
	The biggest concern is the potential for it to cause a rift in rapport, in therapeutic rapport, if something's written in the record that either the patient doesn't quite understand or doesn't like, and that would cause conflict. Not that conflict is necessarily bad, but I think I really pride myself on trying to do the best I can to build rapport with people. The fact that there's this other thing outside the room that has an impact on that can be concerning. I also think you can write your notes in a way that you can be clear about what's going on and what concerns you have but in a respectful way to mitigate that. So I think it's a concern, but for me it's not a huge concern. (1012)
	So many of our veterans feel labeled. If you take someone with various, say, personality disorders or cluster b traits. They read one particular sentence about how you're perceiving their appearance in session and even down to how their affect is. It will completely - I shouldn't say completely – it does often create a lot of additional back work to reestablish rapport, to explain. (1014)
	I'm kind of concerned sometimes if they see the notes, they'll think somehow we're making judgments that are unfounded and will worry that we think badly of them. Because if they aren't here, I'm not here. I'm only here in this job, doing this, because they are here. If they're not included, it really diminishes what I do, because I do patient centered care. So if they don't feel that they're part of it, number one the therapy is not going to work very well - if at all. They're not going to buy in; they're not going to come back; they're not going to do their work; they're not going to reap any of the benefits that I want them to reap. So the more they feel a part of the process, the more they invest in it and feel like they're valued in it. The better results I'm going to get with the work that I do for them. (1017)
	The biggest concern is the potential for it to cause a rift in rapport, in therapeutic rapport, if something's written in the record that either the patient doesn't quite understand or doesn't like or---and that would cause conflict, not that conflict is necessarily bad, but I think I really pride myself on trying to do the best I can to build rapport with people. (1011)
There is the opportunity for a greater sense of trust and transparency, as well as enhanced communication.	I've heard that a couple of times: 'from your charting I could see how much work you've put into it, and I could see that you care about me, and the plans that we come up with - you are hearing the things I want out of our goals and plans.' (1027)

	I think [OpenNotes] offers an opportunity for teachable moments, or it can help increase communication. If they come in here angry about something I wrote, as much as that's a concern, it's also a good thing; they're expressing an emotion to somebody that has been problematic for them to do and is one of the reasons why they are drinking. So I think it can be an opportunity to bring up some stuff about trust and communication and education about whatever issues that you're writing about. Just like it's a concern that this outside entity can affect the therapeutic relationship it's also a benefit. (1012)
	Well I think the major, overarching therapeutic benefit is knowing nothing's being hidden. The being hidden thing is a big issue for everybody, all of us. And I think that's a major – that's one of the first things: they feel that they're trusted enough to see their own information and there's no hiding. (1030)
	I think with the clients that I'm working with it helps to establish that – regain that trust with the VA, like 'Hey here you go, we're being completely open and transparent, there's nothing to hide.' So I think that's been really a benefit. (1013)
	If they come in and say…'Why did you think I was angry?' Then I think that can be something that we can even discuss, so maybe that's a potential benefit. They read how they are coming across to somebody that presumably they trust and have established some rapport. (1009)
	In other words, it facilitates a discussion rather than shutting it down. My greatest fear in therapy is that somebody comes in, tells me something intimately personal and they are not heard or felt like they're not understood. I think when somebody opens the door by saying 'you're not going to chart this' what it allows for is a conversation of 'what do you mean that I'm not going to chart this? What are you concerned about?' It actually allows the person to say 'here's what I'm concerned about.' Generally I think I'm able to navigate with clients in a very human way. 'Here's what I'm hearing from you,' or 'the concerns and the problems that you have about what you fear may be read about you – let's talk about how real that is, or let's talk about your perceptions, or let's talk about my role and how I've charted things before.' It generally is a pretty good conversation. I value it. (1019)
	And for some clients I feel like them being able to read their notes and have those discussions with us about them would actually enhance that trust and that relationship and make things feel more collaborative. (1005)
	I think we are doing our veterans a service if we present the context in a way that is true to their experience. So if you're writing a note in a way that is true to their experience, they won't object. They'll feel like they were understood and heard. It will be enhancing of trust in the treatment relationship, and it could help others to understand the veteran in a way that would make them see them in a more humanized way, a more holistic way. (1018)
**Adjusting to OpenNotes**	
Enhanced patient access adds complexity to the already careful task of writing progress notes.	Patients have brought up things like sexual abuse and then they say, 'I don't want that in the notes because I don't want my provider to see this in there.' There's another one where someone said, 'I smoked meth for 40 years and my wife doesn't know.' And I was like, gosh, do I put this in the note? Because I don't know if he is going to give his wife access to his notes and then see something that was delivered in confidence… (1006)

	Who are we writing these notes for? Is this a record for billing purposes or is this a record for communication with other providers or is this a record that we need to be - ? Often we're taught to document things in a particular way in order to cover ourselves for legal concerns and adding the layer of actually having the client also reading these notes just adds an additional layer of complexity to what you have to think about and how you have to phrase things in your documentation. (1005)
	So I think one concern that clinicians have with OpenNotes, is that now they have to chart for another audience. (1003)
	But when we put a note in here, even within [the electronic health record], it can be read by 10,000 employees. That's why it's been difficult for me. Having the VA, or the veteran and any of his or her associates to read that also, it just makes it feel like, who am I writing this for? Am I writing this for my own clinical charting to understand? Am I writing this as an assessment for public consumption? That's kind of where it comes down. (1006)
	That's the kind of stuff that, in a way, I mean our work is pretty challenging to begin with so it's just another challenge to try to be thoughtful and considerate in that way. Most of us are like that anyway so it's not that much bigger a burden, really. But it's a little extra work to go, 'well wait a minute, before I do this, let me think how this might be interpreted if they are at home reading this.' (1008)
Clinicians feel increased responsibility for protecting their patients from harm resulting from reading their notes, while also feeling the need for protection themselves.	Then again, for me, the onus is on us. We're the ones who are responsible for creating safety. I think that's a big part of this. If OpenNotes were to trigger somebody or create a safety issue, it's still on us to do our best to resolve it in a safe way. It shouldn't be on the person who is sick or war-torn to navigate it. (1011)
	So I just feel like it hinders my ability to work without the feeling – sometimes I feel a little threatened, I feel there is going to be really negative consequences if I write what I'm assessing to be clinically accurate. (1016)
	There was a feeling that providers aren't protected. This is just opening us up, this could potentially create harm for providers… I don't worry about this for the overwhelming majority of the people I serve at all. But there have been some people that over time that you do - it's important to be concerned about. (1021)
	It's going to be that one time, that one veteran is going to read something and is going to hurt themselves. And then I'm going to feel like I have to live with that. (1025)
Discomfort around potential harms may be serving to improve care and documentation.	I think it has this sense of increasing empathy on my part. I really try to see where people are at. And I think when they're coming in here saying, 'this hurt, this is what's written in my record,' it forces us to kind of be in their shoes a little bit when you know they're clicking on that button and seeing what you wrote. (1012)
	When you know that other people are looking at the work that you do, particularly the people who it directly pertains to, then you want to make sure it's the best, it's the most accurate. …So I'd imagine, to some degree, [OpenNotes] could change clinical practice for the better. (1023)
	I think providers being a little bit uncomfortable is kind of important. I think that that keeps us on our toes and makes us more aware when we're writing the note. (1013)

	I'm generally in favor of OpenNotes, because it does require us to be thoughtful and diplomatic and make sure that what we put in the record is justified. (1007)
Clinicians are seeking guidance to reduce likelihood of potential harms.	I would appreciate some clarity on who the audience is, on who I'm writing for. And I think in general, training in this more recovery-oriented and strengths-oriented treatment in general. Moving away from thinking about things, like in the medical model, in terms of problems and thinking about things more as this being a collaborative relationship with their clients. (1005)
	Some sort of agency/VA-type guidance on what's expected to be in a note. What should and shouldn't be left out in order to minimize the risk of the open note problem, or the potential open note problem --- I think at this point we're on our own to do the best we can. It'd be nice to have some guidance on what's okay to do and what's not okay to do. At this point, I don't think there's any sense at all on how to approach that. I don't know that there's really any good answers for it. (1008)
	…it would be really nice for there to be some kind of culture shift with clinicians. So that we're shown what would be helpful as far as documentation goes. So that we have something to base our own standards on. (1025)
	But I would wish for the providers during this transition that there was some education, some chance to get guidance on how to write honest, still-good, accurate clinical notes without reducing people down in a way that's going to be offensive. To remind people, 'you give someone a diagnosis, you tell them first.' It would be like, can you imagine like your doctor diagnoses you with cancer. You don't know that though until you look in your chart. You haven't heard it from the person. You learn it by looking in your chart. Not good. Not good at all. And so just how to be really, really aware of that. Training on just how to remind yourself, remind others. Something that happens a lot. (1026)
	What will our management and leadership support us in writing? What has to be in the note? And where do we have more lee-way? (1013)
	I think that legally [the clinicians] should know that they're covered. That's important. Also that they're supported. That if a Vet gets upset by anything they read that, even with those safeguards in place of the Vet knowing they can talk to their provider or that information being on the website, that they, for the clinician, to know they'll be supported by the VA in their ability to be an independent provider and support them and trust them in their knowledge and their professionalism of what they write in their chart if after they look at it and there's no outrageousness, everything looks right on, you know, you can't, you have to support the clinician. Very important. (1030)
Clinicians are changing their note writing and care delivery in the context of OpenNotes.	How have I adapted? My notes are a lot less detailed now, here. I always have to kind of couch what I'm saying. There's much less detail, much less frankness in my notes now. (1006)
	I do an informed consent about [OpenNotes]. I think it's dangerous, I tell them, 'Look, there's this thing called the blue button. You'll hear about it. You may want to push it. If you do you're going to see your clinical notes. That's fine by me, but understand there's stuff here that I'm going to write what I hear and see and it may be upsetting to you, and you may or may not want to do it, but there's risks associated with it.'

	(1010)
	I feel defensive in the way I document. I tend to limit more of what I will or won't [document], and I'm very concrete and tend to do more quotations. I'm sort of put in this weird defensive position. (1024)
	I think it's unfortunate because I feel like for me writing my notes is the point in my practice where I'm sort of coordinating my thoughts and making those formulations and those judgments and thinking about what I need to do next. By making a conscious effort to not document those as much, I feel like my approach is not necessarily as cohesive. (1005)
	When I know someone or believe in my heart and professional mind that someone is being manipulative, I will not put that in. That's especially true on the side of people with personality disorders who are more often very, very manipulative. So I try to take that – weed that stuff out so it doesn't really look that way. Again, it's kind of blander and more generic. (1008)
	I'm now writing a note that either does not document certain things, that is opaque, is bland and is innocuous and filled with a lot of crap. I'll cut and paste lab values and just fill up shit in there because I don't want to deal with - I don't want to harm the patient and yet there's certain obligatory aspects to being a provider that you're in a rock and a hard place. You have to document certain minimum things, so you fill it up with crap. (1010)
